# Semi-quantitative and structural metabolic phenotyping by direct infusion ion trap mass spectrometry and its application in genetical metabolomics

**DOI:** 10.1002/rcm.4142

**Published:** 2009-08-15

**Authors:** Albert Koulman, Mingshu Cao, Marty Faville, Geoff Lane, Wade Mace, Susanne Rasmussen

**Affiliations:** 1 Agresearch Grasslands Research CentrePrivate Bag 11008, Palmerston North, New Zealand; 2 Biological Mass Spectrometry, MRC, Elsie Widdowson LaboratoryCambridge, UK

## Abstract

The identification of quantitative trait loci (QTL) for plant metabolites requires the quantitation of these metabolites across a large range of progeny. We developed a rapid metabolic profiling method using both untargeted and targeted direct infusion tandem mass spectrometry (DIMSMS) with a linear ion trap mass spectrometer yielding sufficient precision and accuracy for the quantification of a large number of metabolites in a high-throughput environment. The untargeted DIMSMS method uses top-down data-dependent fragmentation yielding MS^2^ and MS^3^ spectra. We have developed software tools to assess the structural homogeneity of the MS^2^ and MS^3^ spectra hence their utility for phenotyping and genetical metabolomics. In addition we used a targeted DIMS(MS) method for rapid quantitation of specific compounds. This method was compared with targeted LC/MS/MS methods for these compounds. The DIMSMS methods showed sufficient precision and accuracy for QTL discovery. We phenotyped 200 individual *Lolium* *perenne* genotypes from a mapping population harvested in two consecutive years. Computational and statistical analyses identified 246 nominal *m/z* bins with sufficient precision and homogeneity for QTL discovery. Comparison of the data for specific metabolites obtained by DIMSMS with the results from targeted LC/MS/MS analysis showed that quantitation by this metabolic profiling method is reasonably accurate. Of the top 100 MS^1^ bins, 22 ions gave one or more reproducible QTL across the 2 years. Copyright © 2009 John Wiley & Sons, Ltd.

Rapid phenotyping of a large number of traits is gaining interest now that genotyping methodologies are becoming quick and relatively inexpensive. Genetic data are of limited use without a correlation to a particular phenotype, and genotyping has so far been primarily related to readily observable phenotypes, such as morphological traits. To exploit genotyping data further correlations need to be established with higher resolution phenotypic data. One of the practical applications of this research area is marker-assisted selection (MAS), which is one of the main biotechnological advancements in plant breeding. Most traits of interest in plant improvement are quantitative in nature, that is, they are influenced by multiple genes and by environmental factors. Regions of the genome that contain genes with measurable effects on a quantitative trait are known as quantitative trait loci (QTL). Molecular markers linked to QTL are identified on genetic maps by QTL analysis and may subsequently be applied in a MAS strategy to screen for individuals in populations that are genetically disposed to producing the desired phenotype, reducing dependence on measurement of the trait itself. This is especially useful when the determination of that specific trait is laborious, prone to error, expensive or the trait is of low heritability. The application of MAS strategies is being used successfully to accelerate the breeding for key economic traits in a number of major crops.^1^,^2^ An area where MAS can be of major importance is in selection for metabolic traits. The measurement of specific levels of metabolites in breeding populations is expensive and time consuming and MAS could potentially speed up breeding for these traits. The use of metabolomic approaches in the identification of QTL can be regarded as a new and promising area in plant breeding.^3^ The combination of genotype and metabolic phenotype opens up the prospects of forward genetics and is a powerful way for establishing relationships between genes and metabolites, the study of which has become known as genetical metabolomics.^4^–^7^

The utility of a metabolic profiling method for phenotyping depends on its speed, cost and coverage, and the precision, accuracy and qualitative information content of the data acquired. Speed is important because of the large sample throughput necessary to provide the scale of data required to establish statistical links between metabolic and genotypic data. Adequate precision and accuracy are essential to make valid observations. The three different strategies that are followed in metabolomics each have their merits and short-comings for metabolic phenotyping. Nuclear magnetic resonance (NMR)-based metabolic profiling can be very reproducible and compatible to large sample sets but only covers a very limited number of major metabolites.^8^ Other scientist have successfully used gas chromatography/mass spectrometry (GC/MS)-based metabolic phenotyping approaches on large sample sets.^9^ However, this method is targeted towards the polar compounds of the primary metabolism and will not provide information on many metabolic traits likely to be of interest. Liquid chromatography/mass spectrometry (LC/MS)- and direct infusion mass spectrometry (DIMS)-based methods are able to measure a wider range of different metabolites. However, the robustness and stability of these methods beyond 100 to 200 runs can be questionable. Metabolic phenotyping using metabolomics techniques is only of interest if it can deliver reproducible data over 1000 or more analyses.^6^

Some metabolic phenotyping strategies assume that each measured signal originates from one single metabolic species.^7^ When low-resolution mass spectrometers with only one unit mass resolution are used several different metabolites can deliver the same signal and even with high-resolution mass spectrometry in DIMS a signal may derive from multiple isomers. The structural homogeneity underlying the measured signals thus needs to be assessed as different metabolites with similar features can generate the same signal. Moreover, limited prior knowledge of metabolite identities in biological samples demands sufficient spectral information to identify or at least classify the observed metabolites.

Methods that perform well in these areas can be applied to metabolic phenotyping of populations for which genotypic data are available, including progenies of specific crosses. In this report, we demonstrate how direct infusion ion trap mass spectrometry (DIMSMS) metabolic profiling identifies structural and quantitative metabolic features that can be used to determine QTL in a plant population. This non-standard approach demanded new data-mining tools (which we developed within R software), enabling the systematic analysis of the data.

In this study we have focused on perennial ryegrass (*Lolium perenne* L.), which is the major forage species in large parts of Europe and Australasia. Like most other temperate and cool grasses this species lives in symbiosis with fungal endophytes,^10^ most commonly *Neotyphodium lolii*. Although these endophytes do not in most cases cause any morphological changes to the plant they play a major role in the use of perennial ryegrass as forage for livestock. Endophytic *N. lolii* fungi produce a range of alkaloids that are crucial for the persistence of the grass in the field, but other alkaloids produced by the fungus can have toxic effects on livestock, such as ryegrass staggers.^11^ It has been shown that host genetics can influence the performance of *N. lolii*, both in terms of fungal biomass and the alkaloid profile.^12^ We were therefore interested to determine QTL for these effects and to determine if there are different QTL for the levels of the different fungal metabolites. This could enable the development of molecular markers for these traits, which would be highly desirable as breeding of grasses for their effect on endophytes is extremely complex and tedious.^13^ Recent studies have shown that there are complex metabolic interactions between the host plant and the endophytic fungus.^14^,^15^ We therefore chose not to limit our analyses to two or three specific fungal alkaloids but to use a rapid DIMS method with an ion trap mass spectrometer enabling both measurement of specific fungal alkaloids and unbiased metabolic profiling of a range of known and unknown metabolites.

A rapid analytical method was required to limit constraints on resources including instrument time. In the first year we used the DIMSMS method as already published, which provided an MS^1^ profile and MS/MS information on ca. 200 major ions.^16^ This method required over 15 min per sample (including controls and blanks), and therefore only two replicate plants were analysed. The extensive MS/MS spectral information yielded by this method assisted identification and the assessment of homogeneity of metabolite composition.^15^ In the second year with extensive MS fragmentation data on the metabolites already in hand we developed an accelerated targeted DIMSMS approach, which enabled three replicate analyses of the 200 plants to be run while collecting fragmentation data on specific ions.

DIMS is not usually advocated as a quantitative method. One of the aims of this study was to compare quantification by DIMS with that by LC/MS/MS and to show that the accuracy is sufficient to determine reproducible QTL across 2 years. We also describe a new method based on direct infusion collecting MS^1^ profile data and targeted MS^2^ and MS^3^ data for selected ions (peramine and ergovaline), which we have designated DIMS(MS). This method yields quantitative information on specific endophyte alkaloids peramine and ergovaline, two well-known metabolites in the symbiosis of ryegrass and endophytes.

## Experimental

### Instrumental

A linear ion trap mass spectrometer (LTQ) coupled to a Surveyor high-performance liquid chromatography (HPLC) system (both Thermo Finnigan, San Jose, CA, USA) was used. Thermo Finnigan Xcalibur software (version 1.4) was used for data acquisition and processing.

### Biological materials

For this experiment an F_1_ mapping population (IxS) from a pair cross between two heterozygous genotypes from two commercial perennial ryegrass (*Lolium perenne* L.) cultivars (‘Grasslands Impact’ × ‘Grasslands Samson’) was used.^17^ The ‘Impact’ parent was infected with a naturally occurring ‘wild-type’ endophyte (*Neotyphodium lolii*) strain. IxS F_1_ mapping population progeny were maternally derived from this parent and, therefore, due to the obligate vertical transmission of endophyte through seed, shared the same endophyte background. Three clonal replicates of 200 genotypes (parents plus 198 F_1_ progeny) were grown outdoors in pots in a randomised complete block at AgResearch Grasslands, Palmerston North, New Zealand. Bulk herbage samples were harvested from two replicates/genotype on 31 March 2005 and from three replicates/genotype on 3 April 2006 and stored at −20°C. Samples were freeze-dried and milled.

### Chemicals

All solvents used for LC/MS were HPLC grade; solvents used for other procedures were of HPLC or analytical grade. A synthetic standard of peramine was supplied by B. Dent (Lower Hutt, New Zealand). Synthetic ergovaline was provided by Dr. F. Smith (Pharmacal Sciences Department, Auburn University, Auburn, AL, USA).

### Extraction

Around 200 mg of each sample was weighed out exactly into screw-topped vials and 1.0 mL of isopropanol/water (1:1) was added. The vials were rotated for 2 h at 30 rpm. After extraction each vial was centrifuged at 13 000 rpm and 100 µL of the supernatant was added to 900 µL of isopropanol/water (1:1) in an HPLC vial. The vials were stored at −20°C until analysis. Aliquots of 100 µL were taken from several randomly selected extracts and combined to give control samples for both years.

### Direct infusion mass spectrometry

DIMSMS analysis was based on the previously described method.^16^ The infusion solvent (0.1% formic acid in H_2_O and 0.1% formic acid in MeCN (1:1)) was pumped at 250 µL min^−1^ and split into a 12 µL min^−1^ flow to the autosampler and a 238 µL min^−1^ flow to a T-junction just in front of the electrospray ionisation (ESI) source. A 40 µL aliquot of each sample was injected in the low flow stream in the autosampler. The sample flowed through the sample loop at 12 µL min^−1^ and joined the T-junction just in front of the ESI source. The flow rate was kept constant for 9 min, after which it was increased to 500 µL min^−1^ for 1 min, switching to 100% MeOH for 1 min followed by 100% isopropanol for 1 min, and then switching back to the original solvent mixture for 1 min. The flow rate was then restored to 250 µL min^−1^ prior to injection of the next sample.

Samples were run in random order. After each 15 samples the control sample was infused, followed by two blank samples. Then the machine was switched to negative mode and all samples were analysed in a comparable way using negative ESI (to be reported elsewhere). During the complete experiment the sample tray of the autosampler was held at 5°C.

The mass spectrometer was set for ESI in positive mode. The spray voltage was 5.0 kV and the capillary temperature 275°C. The ion optics were tuned using paxilline. The flow rates of sheath gas, auxiliary gas, and sweep gas were set (in arbitrary units/min) to 20, 5, and 12, respectively. For the first 1 min after injection no data were recorded; for the period from 1.0 to 1.5 min, MS^1^ spectra only were recorded; from 1.5 to 9.0 min the mass spectrometer was set up in data-dependent mode to collect one MS^1^ spectrum, followed by the isolation (2 mu (nominal mass units)) and fragmentation (35% CE (relative collision energy)) of the most intense ion from the MS^1^ spectrum, followed by the isolation (2 mu) and fragmentation (35% CE) of the most intense ion from the MS^2^ spectrum, and this was repeated in turn for the 25 most intense ions in the MS^1^ spectrum. A new MS^1^ spectrum was then recorded, followed by the repetitive isolation (2 mu) and fragmentation (35% CE) of the 25 most intense ions from that MS^1^ spectrum and their most intense MS^2^ product ions. When an ion with a specific mass was isolated and fragmented for the third time, it was placed on an exclusion list for the duration of the run. In total over 250 MS^2^ spectra of different ions were recorded in an average run.

## DIMS(MS)

The method was similar to the DIMSMS method, using the same setup and solvents but for these experiments a 5 µL aliquot of each sample was injected in the low flow stream in the autosampler and flow rate was kept constant for 2 min after which the same washing steps were used.

The mass spectrometer was set up similarly. For the first 1 min after injection no data were recorded; for the period from 0.6 to 2 min MS^1^ spectra were recorded (100–800 *m/z*), followed by a 4 min section with a cycle of 23 MS^1^ combined with targeted fragmentations for (i) peramine: MS^2^: 248.2 *m/z* (±2 @ 35% CE), MS^3^: 248.2 *m/z* (±2 @ 35% CE); 206.2 *m/z* (±2 @ 35% CE); (ii) ergovaline: MS^2^: 534.3 *m/z* (±2 @ 35% CE), MS^3^: 534.3 *m/z* (±2 @ 35% CE); 516.2 (±2 @ 35% CE).

### LC analysis

Peramine and thesinine-rhamnoside were analysed by LC/MS/MS as previously described.^18^,^19^ Ergovaline was analysed by HPLC-fluorescence.^20^

### Data analysis

The data were extracted and analysed using a modification of the method described by Cao and co-workers.^15^ The ion abundance values for nominal unit mass MS^1^ bins over the range *m/z* 100 to 800 (hereafter referred to as *m/z* bins) were determined for each sample to generate an MS^1^ data matrix for statistical analysis. For the normalisation we used the following strategies. The first step in normalisation was the use of the median intensity for each *m/z* bin.^15^ The median intensity for each bin was then divided by the sum of all the median intensities for the particular sample (similarly to Koulman *et al*.^16^). This largely eliminated the batch effect across the experiment, and this normalisation procedure was sufficient for all the MS^1^ *m/z* bins.

The degree of homogeneity across the samples of the MS^2^ spectra from a given *m/z* bin obtained during the untargeted DIMSMS analysis reveals quantitative or qualitative differences in the composition of the isobaric species within the *m/z* bin. The modified Manhattan distance was used to measure the similarity of sparse MS^2^ spectra derived from a given *m/z* bin.^15^,^16^ Instead of visual inspection, we developed a method to judge whether these MS^2^ spectra are homogeneous based on the cophenetic correlation coefficient (CPCC).^21^ A higher CPCC indicates a higher tendency towards multiple clustering, i.e. the presence of qualitatively different MS^2^ spectra within the set indicating that the signal for a given *m/z* bin is a measure of different metabolites in different samples.

A different procedure was used for the specific measurements of peramine and ergovaline. The MS^3^ intensities were measured constantly during the infusion of the sample, over the course of 2 min. The intensity of the MS^3^ signal showed a near Gaussian curve, which was suitable for integration like a chromatographic peak. We used the LC-Quan option of the Xcalibur software package to integrate the MS^3^ signals of peramine (using the summed signals of the 149.1, 175.2 *m/z* product ions from 206.2 *m/z*) and ergovaline (using the summed signals of the 208.2, 223.2, 268.2, 320.2 *m/z* product ions from 516.2 *m/z*). These data were then normalised relative to the respective signals in the preceding and subsequent control samples in the run. Longer-term variation was then corrected by a second normalisation strategy using the linear regression of the peramine or ergovaline intensity against the run number. This function was considered to describe the decline in the intensity during the whole experiment. Using this function we adjusted the intensity of each measurement to the fitted value.

### QTL analysis

A genetic linkage map of the I × S population^17^ was constructed for QTL analysis using EST-derived simple sequence repeat (SSR)^22^ and sequence tagged site (STS) markers. Briefly, a two-way pseudo-testcross analysis^23^ was used to construct a genetic linkage map based on 188 IxS F_1_ progeny, using the CP (cross pollination) population module in JoinMap® 3.0.^24^ A consensus map based on meioses in both parental genotypes ‘I’ and ‘S’ was estimated, after first checking for conservation of marker locus order between individual parental maps. The final map identified seven linkage groups (LG1–7), equivalent to chromosomes, and is 640 centimorgans (cM) in length with 160 marker loci at a mean density of one locus every 4 cM. Linkage group assignments are consistent with the International Lolium Genome Initiative (ILGI) nomenclature.^25^

QTL analysis was performed using simple interval mapping implemented in MapQTL® 4.0 software.^26^ For each trait, the phenotypic mean value (n = 2 in 2005 and n = 3 in 2006) for each IxS progeny genotype was used for QTL analysis. The software generates a LOD (logarithm-of-odds ratio) score profile across linkage groups for each trait, the LOD score being a statistical test for the presence of a QTL controlling the trait. Peaks that penetrate a pre-assigned minimum LOD threshold indicate the presence of a putative QTL. A LOD threshold of 2.7 for QTL declaration (linkage group-wide significance *P* < 0.05) was chosen based on permutation testing (n = 1000) implemented in MapQTL®. QTL position was described by LOD peak position and 1- and 2-LOD support intervals.

## Results and Discussion

The aim of this study was to develop and apply a method that would rapidly acquire data on as many metabolites as possible with sufficient precision and accuracy for reproducible QTL determination. To handle the scale of the task with available instrument resources we have applied DIMS techniques. As we were aware of the potential limitations of these techniques such as ion suppression, adduct and cluster formation, and the lack of qualitative resolution through chromatography, we investigated the efficacy of the methods in terms of the qualitative homogeneity of the signals and the precision and accuracy of the measurements. We have found these techniques allow for the rapid collection of data on a sufficient scale and with an acceptable level of reproducibility to be used for the detection of QTL.

The analysis was performed over 2 years with two different methods. In the first year the untargeted DIMSMS method was focused on obtaining both quantitative and qualitative data through the collection of MS^2^ and MS^3^ spectra. These spectra facilitated the characterisation and identification of the metabolites occupying the *m/z* bins. They also provided evidence of the homogeneity of a given *m/z* bin across all the samples, which is essential when the *m/z* bin signal intensity is to be used in quantitative analysis. With this information in hand we could focus on the quantitation and we collected only MS^1^ data and MS^n^ on specific ions in the second year. Threshold values were set for precision and accuracy was determined for selected metabolites. Data of sufficient quality were used for QTL discovery across the 2 years.

### Homogeneity

**Figure 1:**
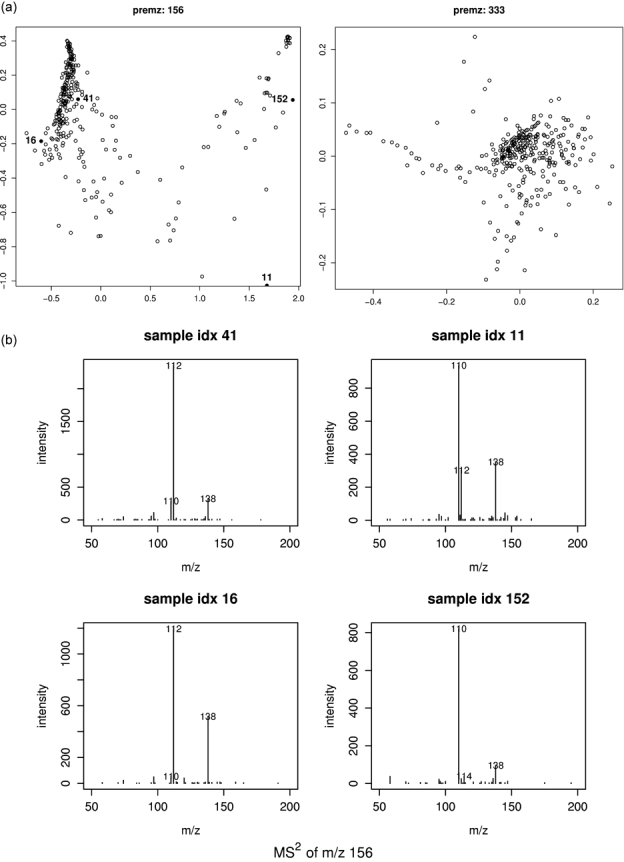
(a) Multidimensional scaling clustering of MS^2^ spectra derived from MS^1^ bins. The 333 *m/z* bin with a CPCC of 0.56 is a homogeneous bin with a few dispersions due to the weak signal. (b) The 156 *m/z* bin with a CPCC of 0.93 is a heterogeneous bin with strong clustering tendency. Four MS^2^ spectra derived from the 156 *m/z* bin are shown; data points corresponding to the sample idx are highlighted as filled black circles in (a).

The large-scale collection of MS^2^ and MS^3^ spectra resulting from DIMSMS allows the qualitative interrogation of the data. The method is based on the modified Manhattan distance as a measurement of the similarity of MS^2^ spectra from ions in a given parent *m/z* bin as previously described in detail.^15^ Clustering was carried out based on the modified Manhattan distance scores between the MS^2^ spectra and the cophenetic correlation coefficient (CPCC) was used to estimate clustering tendency. Multidimensional scaling was used for visual inspection of the homogeneity of the MS^2^ spectra from an *m/z* bin. Bins were considered homogeneous when the CPCC was below 0.9. Of the 337 bins for which valid CPCC scores could be calculated (requiring more than 3 samples with MS^2^ spectra from each bin), 87.2% had a CPCC score <0.9 indicating most bins tend to be homogeneous among samples. Two examples of multidimensional scaling clustering diagrams are shown in Fig. 1. One example is the *m/z* bin 333, with a CPCC of 0.56, which is clearly highly homogeneous across all the samples, showing a very limited dispersion. On the other hand the *m/z* bin 156 with a CPCC score of 0.93 shows a heterogeneous distribution pattern in the multidimensional scaling. Manual inspection of the MS^2^ spectra from four samples that were distant in the multidimensional scaling shows that most likely two sets of ions were present in different relative concentrations. This can be explained by the presence of two different metabolites with the same nominal mass, but at different relative concentrations across the sample set. A CPCC score <0.9 does not establish that an *m/z* bin provides a measure of a single metabolite but it does indicate a consistent set of metabolites across the samples.

We have shown that MS^2^ and MS^3^ spectral information can be used in targeted DIMSMS to quantify metabolites which may be a minor component of an *m/z* bin, as for ergovaline. Further development of both data-mining methods and instrumental technology will be required for this approach to be applied in an untargeted manner.

### Precision

**Figure 2:**
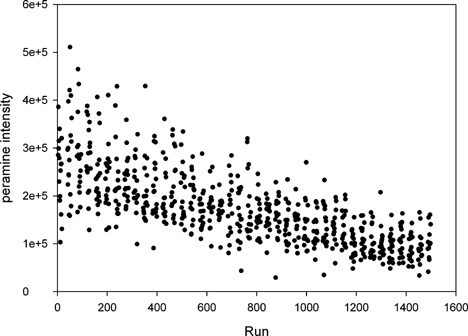
Intensity of peramine signal as measured by DIMSMS before normalisation.

**Figure 3:**
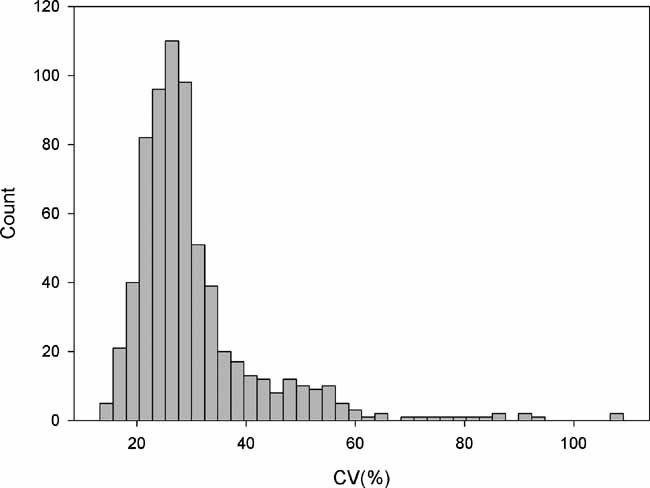
Histogram of the distribution of coefficient of variance values for *m/z* bins in the control sample used in the first year (n = 59).

As previously discussed, the infusion of raw plant extracts affects the overall performance of the mass spectrometer (see Fig. 2). Normalisation of the data is essential to reduce batch effects. In both years a control sample was used to monitor the technical precision and facilitate the normalisation of the data. A straightforward and simple normalisation method was applied as described in the Experimental section. The data on the control sample showed the required precision. The precision of the method can be expressed by the coefficient of variation (CV = 100 × standard deviation/mean) for each *m/z* bin in replicate measurements of a sample. In the first year the DIMSMS method we used focused on collection of MS^2^ and MS^3^ spectra and therefore compromised the number of MS^1^ spectra, which resulted in a less precise method for MS^1^ with a median CV of 29.3% and an average CV of 33.8%. The distribution of the CV values for different *m/z* bins is shown in Fig. 3. In the second year the number of MS^1^ spectra acquired per sample was much higher which dramatically increased the precision yielding a median CV of 18.3%.

For the precise and robust analysis of specific compounds a clear path for method validation exists. However, the development of validated untargeted metabolic profiling is still an area of research and discussion.^27^ The most common approach is the use of a control sample, which is representative for all the samples in the experiment, as discussed above. The decision on the cut-off point for what is considered precise and what not is always arbitrary. For GC/MS data <20% CV has been recommended for quality assurance.^27^ DIMS data are inherently less precise than those from GC/MS. We therefore suggest that initially *m/z* bins with a CV <30% were of sufficient quality to be used in the QTL analysis. For the first year this included over 53% of all the *m/z* bins (see Fig. 3), in the second year this included over 89% of all *m/z* bins. Sufficient precision (CV <30%) and spectral homogeneity of specific *m/z* bins were used as selection criteria for the use of an *m/z* bin in QTL determination.

### Accuracy

**Figure 4:**
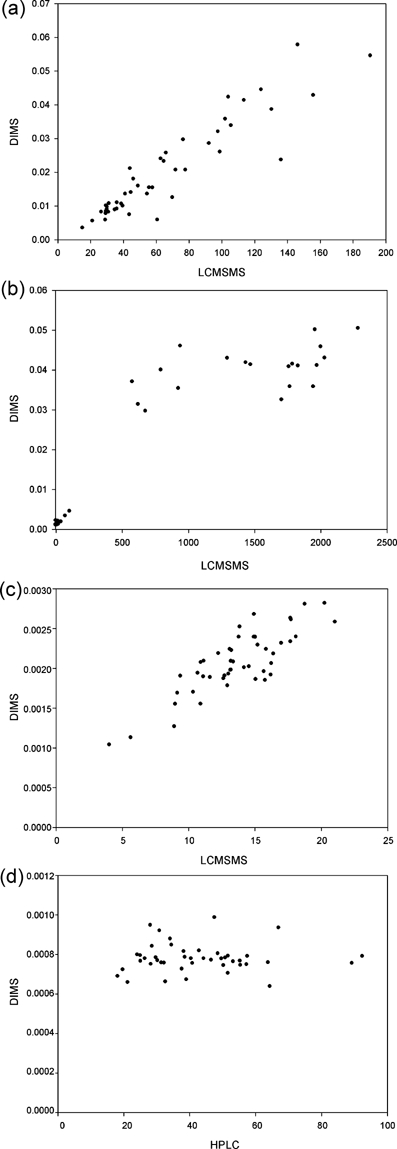
(a) Normalised intensity of the 333 *m/z* bin as measured by DIMS vs. AUC of perloline measured by LC/MS/MS (linear regression R^2^: 0.85). (b) Normalised intensity of the 434 *m/z* bin as measured by DIMS vs. AUC for *E*-thesinine-rhamnoside and *Z*-thesinine-rhamnoside combined measured by LC/MS/MS (linear regression R^2^: 0.86). (c) Normalised intensity of the 248 *m/z* bin as measured by DIMS vs. AUC of peramine measured by LC/MS/MS (linear regression R^2^: 0.70). (d) Normalised intensity of the 534 *m/z* bin as measured by DIMS vs. ergovaline measured by HPLC-fluorescence (linear regression R^2^: 0.00).

A subset of 48 samples randomly chosen from the sample set of the second year was analysed by targeted LC/MS/MS as described previously^18^,^19^ and by HPLC-fluorescence (as described by Spiering *et al*.^20^). This allowed us to determine the accuracy of the semi-quantative data obtained by DIMS with the quantitative data from LC/MS/MS or HPLC. The DIMS values are plotted against the LC estimates for the four compounds analysed: perloline (Fig. 4(a)), *E/Z-*thesinine-rhamnoside (Fig. 4(b)), peramine (Fig. 4(c)) and ergovaline (Fig. 4(d)). The DIMS data for the *m/*z 534 bin corresponding to ergovaline did not show any relationship with quantative data from HPLC-fluorescence. The DIMS data for *E/Z-*thesinine-rhamnoside showed a linear relationship with the LC/MS/MS data over a limited range (Fig. 4(b)). For the other two compounds the correlation between the DIMS and LCMSMS values was linear and considerable based on the R^2^ of the linear regression (see Figs. 4(a) and 4(c)).

**Figure 5:**
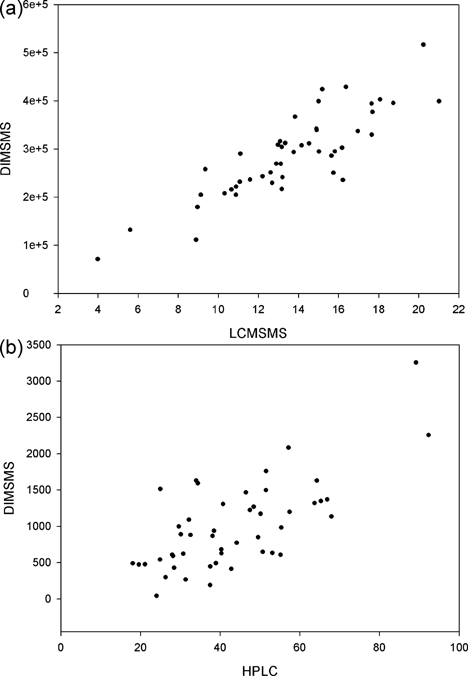
(a) Normalised DIMSMS levels of peramine vs. LC/MS/MS levels of peramine (R^2^: 0.73). (b) Normalised DIMSMS levels of ergovaline vs. HPLC-fluoresence levels of ergovaline (R^2^: 0.49).

In the case of peramine and ergovaline we had also collected MS^3^ spectra by DIMS(MS) and used the summed intensity of selected product ions (149.1, 175.2 *m/z* for peramine and 208.2, 223.2, 268.2, 320.2 *m/z* for ergovaline) to compare with the LC estimates in Fig. 5. For peramine there was a limited improvement in linearity (R^2^ increased from 0.70 to 0.73) but for ergovaline (Fig. 5(b)) the improvement was dramatic (R^2^ increased from 0.00 to 0.49), showing a reasonable correlation between the DIMSMS and LC analysis data.

The compound *E/Z-*thesinine-rhamnoside provided the most abundant signal in a large number of the MS^1^ spectra. This is a recently described alkaloid that is accumulated by several grass species including perennial ryegrass.^19^ By LC/MS/MS it was possible to analyse both the *E*- and the *Z*-enantiomers separately while by DIMS these enantiomers both occupy the 434 *m/z* bin. The ratio between the two enantiomers measured by LC/MS/MS was 1.2 (±0.4). There was a very large difference in concentration between different genotypes. One of the parent plants lacked the ability to accumulate *E/Z-*thesinine-rhamnoside and this was reflected in the progeny where about half of the genotypes did not accumulate *E/Z-*thesinine-rhamnoside, while for other genotypes the 434 *m/z* ion was the highest intensity ion in the mass spectrum. Comparison of the DIMS and LC/MS/MS data (Fig. 4(b)) showed that the dynamic range of the DIMS method is restricted compared to that of the LC/MS/MS method. There is a clear linear relationship between the two data sets until the LC/MS/MS signal exceeds 1300 AUC at which stage the DIMS remains at a plateau between 0.04 and 0.05 (arbitrary units).

For perloline (measured as the 333 *m/z* bin) the linear relationship between the DIMS data and the LC/MS/MS data did not show any saturation as observed for the 434 *m/z* bin and remained linear over the whole scale. The same was true for the peramine signal. Both of these metabolites exist as stable cations, rather than in an acid-base equilibrium as the thesinine-rhamnoside alkaloid. The linearity of the relationship between the LC/MS/MS estimate of peramine levels and those measured by specific DIMSMS was only marginally better than with the 248 *m/z* bin measured by DIMS, which was contrary to what was observed in another ryegrass endophyte association.^15^

This was markedly different for the ergovaline measurements. The levels of ergovaline were very low in these samples, which compromised the precision of even the dedicated HPLC-fluorescence method. Using untargeted DIMSMS the 534 *m/z* bin appeared to comprise largely ions from compounds other than ergovaline. Only MS^3^ data from targeted DIMSMS gave sufficiently ergovaline-specific ions to be used to determine ergovaline levels.

### Speed

An important feature of the method is speed and throughput of the analysis. The actual analysis time per sample was around 12 min in the first year using untargeted DIMSMS and around 5.6 min in the second year using targeted DIMSMS. Additionally, every 15 samples two blanks and two control samples were used which added roughly 25% extra analysis time per sample. The relative short analysis time in the second year enabled us to analyse each genotype in triplicate, which improved the precision.

Sample preparation, especially milling and weighing, are highly time-consuming steps and remain a bottleneck to larger-scale analyses. This step will be unavoidable for any metabolic profiling either using MS, NMR or near-infrared (NIR) spectroscopy, and, of these, MS currently delivers the most information-dense results. An additional separation step in front of MS that does not increase the total analysis time is possible with current sub-2 µm particle columns based on ultra-high-performance liquid chromatography (UHPLC) technology, but will demand sample cleanup to deliver stable analysis able to cope with large sample sets. Also retention time stability across large sample sets is an issue with crude extracts on UHPLC columns (personal observations).

### Ion suppression

The main critique of quantitation by DIMS is that ion suppression is likely to bias results.^28^ Ion suppression is a phenomenon where co-eluting ions influence the ability of an analyte to ionise and its mechanism is poorly understood.^29^,^30^ However, ion suppression has only been studied in detail for LC/MS, with only a very limited number of co-eluting ions. When raw extracts are infused, many thousands of different analytes enter the ESI source at one time, each of which is theoretically capable of suppressing the ionisation of other analytes. It has been argued that ion suppression could render invalid the comparison of DIMS spectra from very different sample types. However, in this study, with samples that are very comparable (maternal sibling perennial ryegrass plants grown together and harvested at the same time), the amount of ion suppression might be reasonably consistent throughout the experiment and therefore not a major problem.

One of the parent plants unexpectedly did not accumulate *E/Z-*thesinine-rhamnoside, and this is apparently a single-gene trait as the progeny were divided into either *E/Z-*thesinine-rhamnoside accumulators with the 434 *m/z* bin as the most intense or non-thesinine-rhamnoside accumulators that usually had the 333 *m/z* bin as the most intense. Thesinine-rhamnoside is a tertiary amine base which ionises extremely well and may therefore cause the suppression of the ionisation of other ions. We estimated the amount of ion suppression by *E/Z-*thesinine-rhamnoside through the calculation of the correlation coefficients for ions that showed negative correlation with the 434 *m/z* bin. The average correlation coefficient of all *m/z* bins (omitting those that are directly (metabolically) related to thesinine-rhamnoside) is −0.29. This can however be mainly attributed to the normalisation, as the median intensity for each bin was then divided by the sum of all the median intensities for the particular sample. In samples with thesinine-rhamnoside present the 434 *m/z* bin attributes to around 5% of the total sum of intensity. In the samples without thesinine-rhamnoside all the other bins are therefore about 5% more intense than in those samples with thesinine-rhamnoside. Therefore, we conclude that thesinine-rhamnoside does not cause any measurable ion suppression. In DIMS with very complex mixtures the presence or absence of one major ion does not necessarily influence the ionisation of other components, which has also been reported in other studies.^31^ Therefore, DIMS may be a more stable and more quantitative platform than generally assumed. This may be further improved by using chip-based nano-ESI, which should be even less effected by ion suppression, but the application of this technology to large sample sets as described in this study has yet to be reported.

### QTL determination

**Figure 6:**
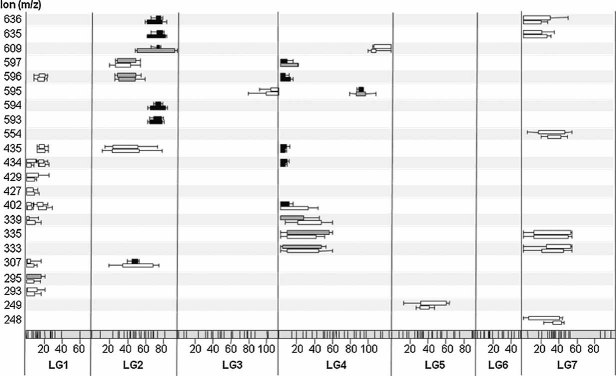
Schematic plot showing the results of QTL interval mapping of selected *m/z* bins. The x axis shows the IxS genetic map consisting of linkage groups LG1–7 presented in tandem. QTL for different ions are positioned along the y axis. Each QTL is represented by a pair of bar plots, with the top one for data collected in 2006 and the bottom one for 2005 (the main block is the 1-LOD support interval, and the error bar is the 2-LOD interval). Colour-coding of QTL bars indicates QTL magnitude: black = logarithm-of-odds ratio (LOD) >10, grey = LOD 4–10, white = LOD 2.7–3.9.

All *m/z* bins were scrutinised for their precision (CV <30%) and homogeneity (CPCC >0.9), which resulted in 246 candidate metabolites. Of these, the top 100 with the best results across 2 years were selected and their QTL were determined. Only QTL that were detected in both years, determined by overlap of 1-LOD support intervals, are reported. This yielded a list of 22 *m/z* bins for which at least one QTL was consistent across 2 years (Fig. 6, Table 2). As indicated in Table 1, once isotopologue ions (and binning variability) are taken account of these correspond to ca. 17 distinct metabolic traits. Our results show that this DIMSMS strategy combining untargeted and targeted methods is very promising for the detection of genetic loci for detailed phenotypes at the metabolic level.

**Table 1:** The *m/z* bins that gave reproducible QTL in the 2 years, with the information on the data quality and identity

*m/z* bin	CV^a^	CPCC^b^	n(MS^2^)^c^	Major MS^2^(MS^3^) fragment ions	Isotopic ion bin^d^	Correlation with isotope^e^	Putative identification
**248**	14	0.50	318	206(175,149), 231	249	0.62	peramine
**249**	11	0.61	315	207 (176, 150), 206(175,149),231,189 (129)			peramine isotopologue + unknown
**293**	17	0.44	311	275(257),171			
**295**	7.0	0.77	17	277, 237, 197			
**307**	9.7	0.45	26	203(161)			
**333**	19	0.56	319	318 (317),317 (315), 289	334,335	0.63, 0.74	perloline
**334**	18	0.34	315	319,318 (316), 290, 289			perloline isotopologue
**339**	5.5	0.69	16	321(303),303			
**402**	8.1	0.66	23	141/142, 124			
**427**	7.4	0.87	6	315, 345			
**429**	5.6	0.60	24	165, 175, 411			
**434**	29	0.83	217	288 (124), 142	435	0.95	thesinine-rhamnoside
**435**	25	0.74	177	289(125,124), 288(124)			thesinine-rhamnoside isotopologue
**554**	23	0.74	23	517, 546	f	0.98	peptide 554.5 2+
**555**	12	0.47	94				peptide 554.5 2+
**593**	22	0.78	319	533 (461)	593,594	0.84, 0.44	unknown
**594**	18	0.59	275	534 (462), 533 (461)			unknown isotopologue
**595**	13	0.59	284	533(461/462)/534			unknown isotoplogue + unknown
**596**	18	0.80	188	288(124), 434, 535	597	0.88	thesinine-rhamnoside-hexoside
**597**	17	0.43	39	288(124), 434, 535			thesinine-rhamnoside-hexoside isotopologue
**609**	20	0.81	223	591(531, 559), 548, 271			
**635**	18	0.39	246	593, 575(533)	636	0.95	unknown
**636**	14	0.64	88	594, 593, 576 (533,534), 575(533)			unknown isotoplogue

a CV: coefficient of variance of the signal from quality control (QC) samples.

b CPCC: cophenetic correlation coefficient based on MS^2^ spectra.

c Number of MS^2^ spectra used for CPCC.

d The related isotopologues.

e The correlation coefficient of this *m/z* bin and the *m/z* bin of the isotopologue.

f The 554 and 555 *m/z* bins are mainly occupied by doubly charged peptide with 554.5 *m/z* that spreads over both bins.

**Table 2:** Details of QTL for 22 ions that were detected in both 2005 and 2006

		QTL peak position (±1-LOD^b^) (cM)		Peak LOD score		PVE^c^ (%)	
Ion (*m/z*)	LG^a^	2005	2006	2005	2006	2005	2006
**248**	7	36.4 (33.2–43.1)	31.9 (6.1–40.4)	4.4	5.8	10.9	14.5
**249**	5	31.2 (28.5–38.3)	37.5 (28.9–57.4)	2.8	2.9	6.8	7.3
**293**	1	1.5 (0.1–9.0)	5.0 (0.7–12.1)	3.3	3.4	9.1	8.3
**295**	1	1.5 (0.4–8.0)	1.5 (0.2–16.7)	3.3	4.2	9.1	11.6
**307**	1	1.5 (0.4–8.0)	1.5 (1.0–4.6)	2.9	3.3	8.3	9.5
	2	46.3 (34.5–68.3)	48.0 (45.0–51.5)	2.9	11.0	9.1	25.0
**333**	4	19.7 (7.0–42.6)	19.7 (2.0–45.6)	3.9	7.9	10.2	19.8
	7	37.0 (20.7–45.2)	37.0 (25.7–53.0)	3.1	3.4	7.9	8.4
**335**	4	19.7 (7.0–39.4)	37.4 (7.1–54.3)	3.2	5.3	8.3	13.1
	7	37.0 (12.2–49.8)	37.0 (12.2–53.0)	3.0	3.4	7.7	8.5
**339**	1	1.5 (0.0–10.0)	1.5 (0.0–4.3)	2.8	2.8	8.1	7.1
	4	30.8 (19.3–45.6)	19.2 (0.0–25.6)	2.9	5.4	7.9	13.5
**402**	1	5.0 (0.7–6.5)	1.5 (0.4–5.5)	3.6	2.9	9.0	8.1
	1	16.1 (13.2–22.2)	16.1 (12.2–19.0)	2.8	2.8	8.2	7.6
	4	0.0 (0.0–30.8)	0.0 (0.0–9.7)	3.3	13.4	10.1	28.4
**427**	1	1.5 (0.2–9.1)	1.5 (0.6–7.3)	2.8	3.1	7.6	9.3
**429**	1	1.5 (0.0–9.0)	1.5 (0.5–13.2)	3.6	2.8	10.0	7.9
**434**	1	1.5 (0.0–5.1)	1.5 (0.0–10.2)	3.3	2.8	9.8	7.2
	1	16.1 (14.0–20.8)	16.1 (13.6–19.5)	2.8	2.8	8.1	8.6
	4	0.0 (0.0–5.1)	0.0 (0.0–7.0)	41.4	51.8	86.2	89.4
**435**	1	16.1 (11.8–21.0)	16.1 (14.0–20.4)	2.8	2.8	8.1	8.0
	4	0.0 (0.0–5.1)	0.0 (0.0–7.0)	42.2	53.6	86.6	89.8
**554**	7	38.7 (27.0–41.8)	38.7 (17.2–46.2)	3.0	3.1	7.4	7.8
**593**	2	68.3 (64.6–78.9)	73.9 (69.5–78.0)	15.2	15.0	33.2	34.9
**594**	2	73.9 (65.5–78.9)	73.9 (71.7–78.0)	10.8	15.1	28.7	35.4
**595**	3	113.2 (99.2–113.2)	113.2 (103.9–113.2)	2.7	3.9	6.5	10.6
	4	91.3 (84.8–95.0)	91.3 (87.1–91.9)	4.5	22.1	15.4	44.2
**596**	1	16.1 (20.0–23.0)	16.1 (20.5–23.8)	2.8	2.8	8.6	8.6
	2	48.0 (30.3–48.5)	48.0 (28.6–49.7)	5.2	6.1	13.2	14.9
	4	0.0 (0.0–10.9)	0.0 (0.0–5.2)	17.8	25.2	35.2	46.6
**597**	2	48.0 (30.3–48.5)	48.0 (28.6–49.7)	3.5	7.4	9.8	18.5
	4	0.0 (0.0–19.3)	0.0 (0.0–7.6)	6.6	16.8	19.2	27.5
**609**	2	63.5 (45.0–92.9)	73.9 (73.0–75.7)	4.6	24.2	12.3	47.7
	4	104.6 (101.3–106.2)	107.2 (104.6–123.7)	2.8	2.9	7.0	7.8
**635**	2	68.3 (62.9–82.7)	73.9 (73.1–79.4)	15.3	20.7	33.2	42.3
	7	7.2 (0.0–26.1)	0.0 (0.0–20.5)	2.9	3.0	11.3	17.2
**636**	2	63.5 (61.8–78.9)	73.9 (72.5–77.2)	12.5	20.5	30.2	42.5
	7	0.0 (0.0–19.9)	0.0 (0.0–30.0)	3.2	2.8	12.8	7.7

a LG = linkage group.

b LOD = logarithm-of-odds score.

c PVE = proportion of the trait phenotypic variance explained by the QTL.

The QTL determined for peramine (248 *m/z*), *E/Z-*thesinine-rhamnoside (434 *m/z*) and perloline (333 *m/z*) using the targeted DIMS(MS) method were consistent with the results obtained using untargeted DIMSMS to analyse the same progeny grown in the previous year (2005). Although further careful analysis of data quality from MS-based high-throughput experiments is warranted,^32^ the comparison of LC/MS/MS with DIMSMS, preliminary statistical evaluation and QTL analysis provided evidence of consistency of the DIMSMS analysis and that the results were reproducible with sufficient accuracy. The ability to reproduce the QTL for *E/Z-*thesinine-rhamnoside (434 *m/z*) shows that a CV of just below 30 gives sufficient precision for reproducible QTL discovery.

As noted in Table 1, several of the *m/z* bins for which QTL are shown in Fig. 6 are clearly isotopologues. These can be recognised by their 1 Da difference and identical QTL, and by the observation of product ions differing by 1 Da in the MS^2^ and MS^3^ spectra. Well-defined QTL, one of which was in common, were identified for two unidentified compounds observed as monoisotopic and isotopologue pairs (593 and 594 *m/z*; 635 and 636 *m/z*). The 248, 333 and 434 *m/z* bins are discussed above and, in the cases of perloline and *E/Z-*thesinine-rhamnoside, isotopologue ions also map to the same QTL. *E/Z-*Thesinine-rhamnoside can also be further glycosylated^19^ and the MS/MS data are consistent with the assignment of the 596 and 597 *m/z* bins to the corresponding glycoside. The 554 and 555 *m/z* bins have previously been assigned to an unknown peptide (554.5 *m/z* doubly charged).^15^ By contrast, the 249 *m/z* bin shows a reproducible QTL quite distinct from the peramine QTL. The CPCC score suggests this bin is essentially homogenous across the samples, but comparison of the MS/MS data for peramine and the 249 *m/z* bin (Table 1) indicates the 249 *m/z* bin includes a signal from the peramine isotopologue, and another species (MS^2^ product ion 189 *m/z*; MS^3^ > 129 *m/z*). Manual interrogation of the data showed the 249 *m/z* bin is on average more intense than the 248 *m/z* bin indicating that the other metabolite detected in this *m/z* bin dominates the signal of the peramine isotopologue of 249 *m/z* (in theory 15% of the peramine signal). The identities of this and the metabolites detected in the other *m/z* bins shown in Table 1 and Fig. 6 have yet to be confirmed.

## CONCLUSIONS

Direct infusion ion trap mass spectrometry is a rapid method that can be used to profile large numbers of samples with sufficient precision and accuracy for QTL discovery across a large range of metabolites. Although the method showed a clear run sequence effect, with appropriate controls and normalisation this systematic error could be corrected. The DIMS data have been shown to be semi-quantitative without apparent major ion suppression effects and has provided data on the variation of some known metabolites within the mapping population, and a large range of metabolites for which MS^2^ and MS^3^ spectra are available for identification or classification. Concurrent targeted MS^3^ analysis allowed simultaneous quantitation of important low abundant metabolites. The data have been successfully used for QTL discovery and a subset of the QTL was confirmed with metabolite data from successive harvests, showing that the analytical method is robust.
